# The Army Canteen

**Published:** 1913-04

**Authors:** R. H. L. Bibb


### The Army Canteen.

Dr. Maus, chief surgeon of the Eastern Division of the United States Army, publishes an exceedingly interesting article in the *New York Medical Record* for February 22, 1913, in which he supports, from official data, his refutation of the charges that abolition of the army canteen, made by the pro-canteenists, has increased sickness, inefficiency, drunkenness, desertions, diseases, insubordination and vice in the army, and has multiplied the number of saloons and brothels contiguous to army posts.

Dr. Maus quotes from the surgeon general that "statistics do not show that absence of beer from the canteens has increased the admissions to sick reports or the ratio of constantly sick," and claims that the report of that functionary for 1912 discloses the fact that the health of the army was never better; that the ratio for sickness for 1911 was 7.6944 per 1000, and 7.6606 for 1910, the lowest ratio in the history of the army, while during the canteen period it was 10.90 per 1000, with a yearly average of 12.46, and that during the period of a beerless canteen the non-efficiency of the army has been 33 per 1000, and 42 during the flourishing days of beer-drinking canteens. The admission rates due to alcoholism during the beer-drinking period was 46 per 1000, and only 20 since its abolition, while desertions have steadily decreased in proportion, until it was actually less in 1911 than at any time during the last ninety years, and he challenges any one to truthfully refute these facts and figures.

Concerning diseases due to vice, Dr. Maus, with an experience of nearly forty years, claims that prior to 1901 but little attention was paid to diseases from this source and that 60 per cent, at least, of soldiers thus afflicted received no official attention, and that the records during the canteen period bearing on this phase of the subject are extremely faulty and incomplete, but, judging from his own large experience and that of very many of his fellow surgeons, he believes they are less than at any time during the canteen period, and that if the recorded rate of such diseases during the time of the beer canteens, that of 75 to 85 per 1000, was multiplied by three it would be much nearer the actual figures, and that it would give a rate of from 225 to 250 per 1000, as against the present rate of 163. During the canteen period, in 1890, the rate at Columbus Barracks, Ohio, was 462 per 1000, and at many other posts during that year it was as high as 250 per 1000.

Dr. Maus claims that hundreds of cases of syphilis carried on the sick reports of today were contracted prior to enlistment and were disclosed only by the Wassermann reaction, and quotes Major Howard, of Jefferson Barracks, Missouri, that 50 per cent of venereal cases treated at that point were ante-enlistment infections. Jefferson Barracks is one of the principal recruiting stations, through which thousands of recruits yearly pass.

Maus denies that abolition of the canteen has multiplied the number of saloons near army posts and cites Fort Sam Houston, Fort Hamilton, Fort Wingate, Brooklyn, New Mexico, etc., where every street surrounding the posts contained saloons. He regards the beer canteen as a kindergarten for the young recruit and training school for the alcoholic habit, where hundreds of the bravest and best of officers and men are cut down and rendered unfit in the prime of life, many being forced to drink to escape the gibes and jeers of their companions.

Beer drinking has the same effects, morally and physically, upon the American soldier that it has upon the American civilian, and produces the same pathological conditions that are produced by other alcoholic beverages, but excites the venereal appetite more than the others excite it. Hence it is that a few glasses of beer at the army canteen, a few more in nearby saloons, the soldier's animal instincts are aroused, and he at once marches direct to the red-light district—sometimes as far as six miles away—where he is often drugged, robbed, infected and oftentimes murdered outright—facts which are well known to the War Department as they are to the few braided and buckled officials who, with their wives and some equally misguided civilians, are clamoring for the government to restore this prolific source of inefficiency, prostitution, disease, death and perdition, as a constant temptation to the men on whom the nation must rely in time of danger, and that, too, in face of the fact that manufacturers, merchants, bankers, farmers, civic and public-utility corporations and even liquor dealers themselves are barring from employment men upon whom the drink habit has impressed even its slightest imprint. No one, be he soldier or civilian, matron or virgin, young or old, whose system is daily polluted with alcohol, even in minute quantities, is in a normal condition, and, *pari pasu,* with increase in the quantity ingested does such an one become less normal and less capable.

R. H. L. BIBB.

